# Design and Implementation of Low-Complexity Multiple Symbol Detection Algorithm Using Hybrid Stochastic Computing in Aircraft Wireless Communications

**DOI:** 10.3390/e27040359

**Published:** 2025-03-28

**Authors:** Yukai Liu, Rongke Liu, Kairui Tian, Zheng Lu, Ling Zhao

**Affiliations:** 1 School of Electronic and Information Engineering, Beihang University, Beijing 100191, China; ykliu@buaa.edu.cn (Y.L.); philtian@buaa.edu.cn (K.T.); htluzheng@buaa.edu.cn (Z.L.); zhaoling@buaa.edu.cn (L.Z.); 2 Shenzhen Institute of Beihang University, Shenzhen 518063, China

**Keywords:** multiple symbol detection, hybrid stochastic computing, stochastic adder, pipeline, aircraft wireless communications

## Abstract

The Multiple Symbol Detection (MSD) algorithm can effectively lower the demodulation threshold in Frequency Modulation (FM) technology, which is widely used in aircraft wireless communications due to its insensitivity to large Doppler shifts. However, the high computational complexity of the MSD algorithm leads to considerable hardware resource overhead. In this paper, we propose a novel MSD architecture based on hybrid stochastic computing (SC), which allows for accurate signal detection while maintaining low hardware complexity. Given that the correlation calculation dominates the computational load in the MSD algorithm, we develop an SC-based, low-complexity unit to perform complex correlation operations using simple hardware circuits, significantly reducing the hardware overhead. Particularly, we integrate a flexible and scalable stochastic adder in the SC-based correlation calculation, which incorporates an adjustable scaling factor to enable high distinguishability in all possible correlation results. Additionally, for the symbol decision process of the MSD algorithm, we design a binary computing-based pipeline architecture to execute the computing process serially, which leverages the inherent low update rate of SC-based correlation results to further reduce the overall resource overhead. Experimental results show that, compared to an 8-bit quantization MSD implementation, our proposed hybrid SC-based MSD architecture achieves a comparable bit error rate while reducing the hardware resources to 69%, 45%, and 36% of those required for the three-, five-, and seven-symbol MSD algorithms, respectively.

## 1. Introduction

**Figure 1:**
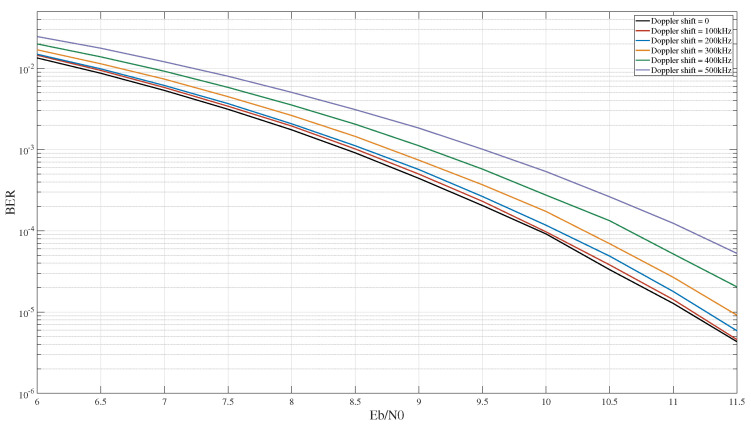
The BER of 3-symbol MSD algorithm under different Doppler shifts.

Considering the global seamless coverage of 6G networks, high-speed aircraft require stable wireless communication connections to guarantee the effective execution of diverse missions [1,2]. Due to its insensitivity to large Doppler shifts, Frequency Modulation (FM) technology has been widely used in aircraft communications standards [3] such as IRIG-106 [4] and DVB-S2 [5]. To ensure the quality of signal demodulation, the Multiple Symbol Detection (MSD) algorithm [6] is a key signal processing technique used in the FM receiver. Figure 1 shows the bit error rate (BER) performance of the three-symbol MSD algorithm under different Doppler shifts, from which it can be seen that, when the symbol rate is 10 Msps and the Doppler shift is 100 kHz, the BER loss is almost negligible. Therefore, FM technology exhibits remarkable superiority in resisting Doppler shifts.

The MSD algorithm can enhance the signal detection performance by leveraging the continuity between different symbols based on the maximum likelihood principle. The essence of the MSD algorithm lies in its ability to holistically process multiple consecutively received symbols as a complete unit, transcending the limitation of relying solely on a single acquired symbol [7]. MSD algorithms can overcome the BER loss of traditional single-symbol detection algorithms in low signal-to-noise ratio (SNR) scenarios, effectively reducing the demodulation threshold of the receiver [8,9].

However, due to the need to calculate correlation results between the received signal and all possible local signals, a large number of multiplication and addition operations are required, leading to considerable computational complexity [10]. Therefore, this study starts from fundamental computational units such as multipliers and adders, and leverages stochastic computing (SC) to mitigate the computational complexity of MSD algorithms.

As a novel computational paradigm, SC has been proposed to effectively reduce the complexity of hardware circuits and improve their computational efficiency [11,12,13], and has been applied to a wide variety of applications such as neural networks [14,15,16], image processing [17,18,19], and channel coding [20,21,22].

SC uses random bits to encode binary numbers [23]. Information is contained in a series of random bits, where the probability of logical 1 represents the target values. In SC, as the computational process is transformed into operations on single-bit data streams, arithmetic circuits exhibit exceptional simplicity [24]. Specifically, for unipolar input data, the multiplier circuit is an AND gate, and for bipolar input data, the multiplier is an XNOR gate. This simple structure presents significant advantages over alternative approximation paradigms, such as fixed-point arithmetic [25,26] or neural-based approximations [27,28,29].

However, the correlation between two stochastic sequences in the SC context has a pivotal influence on the computational outcomes [30]. This correlation can potentially necessitate a longer sequence to ensure the reliability of the computational results. Consequently, this may compromise the real-time performance of the system, posing challenges in terms of timeliness and responsiveness.

Due to its ability to enhance the computational efficiency of SC, the hybrid SC has attracted the attention of researchers [31,32,33,34,35]. SC exhibits low resource consumption, yet demonstrates inferior computational accuracy compared to binary computing. Conversely, binary computing achieves higher computational precision at the expense of higher computational complexity compared to SC. Hybrid computation can leverage the unique advantages of both stochastic and binary computing, while simultaneously addressing their respective limitations. Through integrating these two computing paradigms, hybrid computation offers a comprehensive solution that maximizes the benefits and minimizes the drawbacks of each individual method.

In this study, we delve into the computational process of the MSD algorithm, encompassing two distinct stages: correlation calculation and symbol decision. The correlation calculation necessitates substantial complex multiplication and addition operations, constituting the major computational load in the entire MSD algorithm. To alleviate the resource overhead, the SC is utilized upon conducting correlation calculations due to its characteristic simple computing circuit. Moreover, the correlation calculation requires summing a series of data. Given the constraint in SC that the result must fall within the interval of 0 to 1, this study introduces a stochastic adder with an adaptable scaling factor to guarantee that the summation results meet the specified range. Furthermore, considering the need to identify the maximum value among all correlation results for the subsequent symbol decision, selecting an appropriate scaling factor in stochastic addition also ensures discrimination between different computational results, thereby guaranteeing the detection performance of the entire MSD algorithm.

When it comes to symbol decision, the modulus of the correlation results must be calculated. If the SC is adopted in this process, a new random sequence should be generated based on the corresponding results. However, the complete independence of the regenerated random sequence from the original sequence cannot be guaranteed. This will result in decreased accuracy of the computational results, thereby leading to a loss in the detection performance of the MSD algorithm. Considering the limitations of SC, binary computing is adopted for the symbol decision in the MSD algorithm. Additionally, the errors in binary computing are due to minor deviations from data truncation during the bit quantization process. Therefore, the adoption of binary computing becomes a viable option in the process of symbol decision. Due to the requirement of a certain computation time to ensure the convergence of results in SC-based correlation calculation, there is ample computation time for the binary-computing-based symbol decision. Through meticulously designing a pipeline architecture, the hardware resources needed for binary computing can be significantly reduced, thereby optimizing the overall computational efficiency.

This study makes the follow contributions:

- A hybrid stochastic computing architecture for the MSD algorithm is proposed. By utilizing SC and binary computing at various computational stages in the MSD algorithm, based on the intrinsic properties of the different computing paradigms, the proposed method significantly reduces hardware resource consumption while maintaining the signal detection performance.
- Considering the constraints on the range of data values in SC, a scalable stochastic adder is proposed to achieve the summation of different sequences. This adder can permit different scaling factors, thereby facilitating the selection of optimal parameters tailored to various input conditions. This ensures that the computational results do not exceed the prescribed limits, while simultaneously guaranteeing computational accuracy.
- Due to the low update rate of the SC results, a pipeline architecture is proposed to maximize the utilization of computing time for the symbol decision of the MSD algorithm. The fully serial computation approach is employed to minimize the resource overhead in binary computing.

The rest of this paper is organized as follows. Section 2 introduces the related works, including those on the MSD algorithm, FM signal demodulator, and the fundamental computational methods of SC. Section 3 describes the hardware circuits of the basic SC units and presents an architecture for the proposed hybrid computing scheme that integrates stochastic and binary computing. Section 4 analyzes the computational accuracy and hardware resource consumption of the basic SC unit. Section 5 assesses the computational accuracy, BER performance, and hardware resource of the MSD algorithm based on hybrid SC. Section 6 summarizes the key points discussed in this paper.

## 2. Related Work

### 2.1. MSD Algorithm

The MSD algorithm utilizes the maximum likelihood criterion to decide the received signal within the observation time, with the aim of minimizing the error rate in detecting the transmitted symbol.

The sequence of transmitted symbol can be expressed as

(1) $$s=\left[s_{1},s_{2},s_{3},\cdots ,s_{n}\right]$$

Taking into account the impact of noise, a potential symbol sequence obtained from demodulation can be expressed as

(2) $$\tilde{r}=\left[\tilde{r_{1}},\tilde{r_{2}},\tilde{r_{3}},\cdots ,\tilde{r_{n}}\right]$$

Therefore, the BER equation can be expressed as

(3) $$P_{e}=P\left(\tilde{r}\neq s|r\left(t\right)\right)=1-P\left(\tilde{r}=s|r\left(t\right)\right)$$

where r(t) is the received signal.

According to Equation (3), in order to minimize the BER in the demodulator, the receiver must maximize the value of P(s∣r(t)). Based on Bayes’ Rule, P(s∣r(t)) can be rewritten as

(4) $$P\left(s|r\left(t\right)\right)=\frac{P(sr(t))}{P(r(t))}=\frac{P(r(t)|s)P(s)}{P(r(t))}$$

In Equation (4), P(r(t)) remains invariant regardless of the decision outcome. It is commonly presumed that the transmitted symbols adhere to uniform probability distribution, whereby the value of P(s) remains constant with variations in the decision outcome. To determine the s that maximizes P(s∣r(t)), it suffices to identify the s that maximizes the P(r(t)∣s). As the noise is additive white Gaussian noise, P(r(t)∣s) can be expressed as

(5) $$P\left(r\left(t\right)|s\right)=\frac{1}{{\left(2\pi N_{0}\right)}^{n/2}}\exp\left[-\frac{1}{2N_{0}}\sum_{k=1}^{n}{\left(r_{k}-s_{k}\right)}^{2}\right]$$

Taking the logarithm of Equation (5), we obtain

(6) $$ln\left(P\left(r\left(t\right)|s\right)\right)=-\frac{n}{2}ln\left(2\pi N_{0}\right)-\frac{1}{2N_{0}}\sum_{k=1}^{n}{\left(r_{k}-s_{k}\right)}^{2}$$

In order to maximize the P(r(t)∣s), it is necessary to minimize $\sum_{k=1}^{n}{\left(r_{k}-s_{k}\right)}^{2}$, which can be rewritten as

(7) $$\sum_{k=1}^{n}{\left(r_{k}-s_{k}\right)}^{2}=\sum_{k=1}^{n}r_{k}^{2}-\sum_{k=1}^{n}{\left(2r_{k}s_{k}\right)}^{2}+\sum_{k=1}^{n}s_{k}^{2}$$

where $\sum_{k=1}^{n}r_{k}^{2}$ and $\sum_{k=1}^{n}s_{k}^{2}$ can be considered to be constant values. Consequently, the fundamental principle of the MSD algorithm is minimizing the BER of the demodulation at the receiver by identifying the symbol sequence $\tilde{r}$ that corresponds to the maximum of $\sum_{k=1}^{n}{\left(2r_{k}s_{k}\right)}^{2}$.

### 2.2. FM Demodulator Based on MSD Algorithm

The intermediate frequency (IF) FM signal can be expressed as

(8) $$r_{IF}\left(t\right)=\cos\left(2\pi f_{c}t+k_{f}\sum_{0}^{n}s\left(k\right)t+\theta_{0}\right)$$

where $f_{c}$ is the carrier frequency, $\theta_{0}$ is the initial phase, and $k_{f}$ is the modulation factor.

By down-converting the received signal, the baseband signal can be expressed as

(9) $$r_{base}\left(t\right)=\cos\left(k_{f}\sum_{0}^{n}s\left(k\right)t+\theta_{e}\right)+j\sin\left(k_{f}\sum_{0}^{n}s\left(k\right)t+\theta_{e}\right)$$

where $\theta_{e}$ is the phase error between the received signal and the local carrier.

The local signal in the receiver can be expressed as

(10) $$m\left(t\right)=\cos\left(k_{f}\sum_{0}^{n}m\left(k\right)t\right)-j\sin\left(k_{f}\sum_{0}^{n}m\left(k\right)t\right)$$

Performing correlation operations between the down-converted baseband signal and the local signal, the result can be expressed as

(11) $$r_{base}\left(t\right)\times m\left(t\right)=\cos\left(k_{f}\sum_{0}^{n}s\left(k\right)t-f\sum_{0}^{n}m\left(k\right)t+\theta_{e}\right)+j\sin\left(k_{f}\sum_{0}^{n}s\left(k\right)t-f\sum_{0}^{n}m\left(k\right)t+\theta_{e}\right)$$

Considering that the subsequent decoding module requires soft information from the demodulation results, the soft output can be represented as

(12) $$llr\left(t\right)=\max_{m=0}|r_{base}{\left(t\right)\times m\left(t\right)|}^{2}-\max_{m=1}{\left|r_{base}\left(t\right)\times m\left(t\right)\right|}^{2}$$

**Figure 2:**
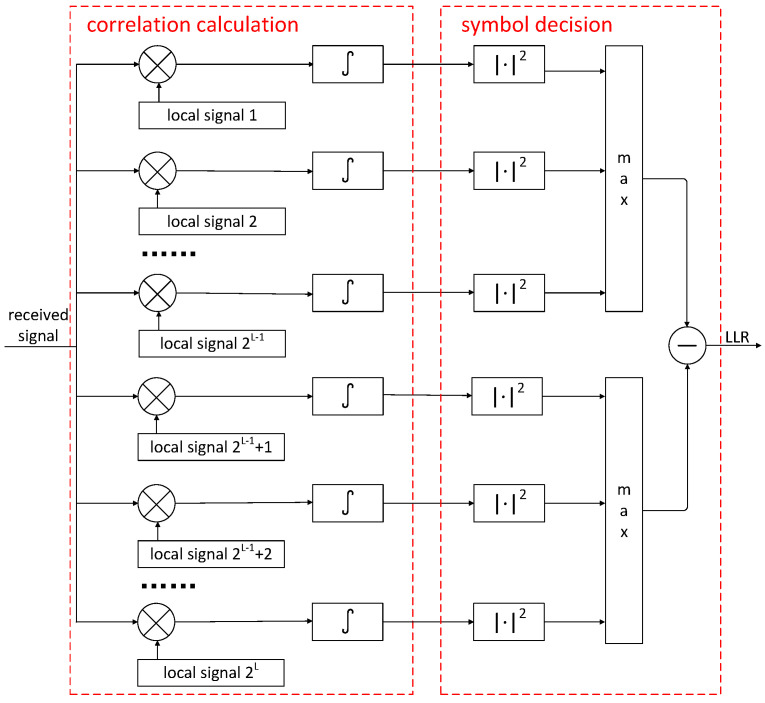
FM demodulator based on MSD algorithm.

Figure 2 illustrates the fundamental structure of the FM signal demodulator based on the MSD algorithm. The entire receiver requires $2^{L}$ local signals to perform the correlation calculation with the received signal, where *L* is the symbol length within the MSD observation window.

Assume that the received signal consists of *N* symbols, where the number of sampling points per symbol is $N_{s}$. Therefore, for one symbol demodulated by the MSD algorithm, $N_{s}\times L$ sampling points must be used in the computational process.

When demodulating one symbol using the MSD algorithm, the number of real multiplications that are required to be executed in the correlation calculation is

(13) $$Mul_{1}=4\times L\times N_{s}\times 2^{L}$$

As both the received signal and the local signal are complex data, and one complex multiplication requires four real multiplications, a factor of four is used in Equation (13).

In the correlation calculation, the number of real additions that must be executed is

(14) $$\begin{array}{cc} Add_{1} & =2\times L\times N_{s}\times 2^{L}+2\times \left(L\times N_{s}-1\right)\times 2^{L}\\ \; & =\left(4\times L\times N_{s}-2\right)\times 2^{L} \end{array}$$

Note that completing one complex multiplication requires not only four real multiplications but also two real additions, and the summation of $N_{s}\times L$ complex data necessitates $N_{s}\times L-1$ complex additions. Moreover, one complex adder requires two real additions. Thus, Equation (14) is obtained.

Similarly, in the symbol decision, the number of real multiplications that are required to be executed is

(15) $$Mul_{2}=2\times 2^{L}$$

and the number of real additions that need to be executed is

(16) $$Add_{2}=2^{L}$$

Therefore, the number of real multiplications required for demodulating one transmitted symbol using MSD is

(17) $$Mul=Mul_{1}+Mul_{2}=\left(4\times L\times N_{s}+2\right)\times 2^{L}$$

The number of real additions is

(18) $$Add=Add_{1}+Add_{2}=\left(4\times L\times N_{s}-1\right)\times 2^{L}$$

The symbol length *L* of the MSD algorithm is generally set to three, five, or seven. Table 1 demonstrates the computational complexity of the MSD algorithm, where $N_{s}$ is set to four. As the observation window of the MSD algorithm increases, its computational complexity grows exponentially.

### 2.3. Stochastic Computing

SC, as an innovative computing paradigm, can represent a numerical value between 0 and 1 through the use of random bit streams. The probability of the logical 1 appearing within a random sequence corresponds to its representation result; for instance, the random sequence 01100010 contains five zeros and three ones; hence, the probability value of this sequence is determined to be three-eighths. As probability values are characterized by finite-length sequences, errors are inevitably present. In accordance with the Law of Large Numbers, as the length of a random sequence increases, the precision of its probability value tends to increase.

The conversion between binary data and random sequences is important in the realm of SC. This is primarily accomplished through stochastic number generators (SNGs), and the conversion equation can be expressed as

(19) $$X\left(t\right)=\left\{\begin{array}{c} \begin{array}{cc} 1 & x\geq n(t) \end{array}\\ \begin{array}{cc} 0 & x<n(t) \end{array} \end{array}\right.$$

where X(t) is the stochastic sequence, n(t) is the random number, and *x* denotes the binary data to be converted.

**Figure 3:**
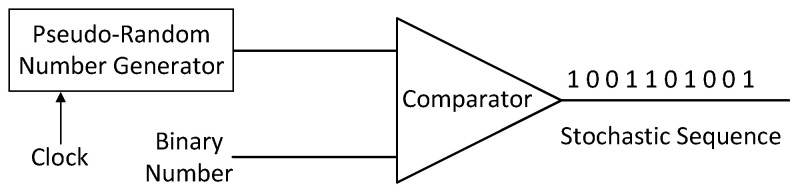
Stochastic number generator.

Figure 3 illustrates the structure of the SNG, which encompasses a pseudo-random number generator and a comparator. Through comparing the input binary data with the generated random numbers, the binary data are subsequently transformed into a stream of single-bit data. Furthermore, SC requires a stochastic number converter (SNC) to transform random bit sequences into binary data. The equation for the SNC can be expressed as

(20) $$x=\frac{1}{L}\sum_{t=1}^{L}X(t)$$

where *L* is the sequence length. The SNC can be implemented through a counter in hardware circuits. When the logical 1 appears in the random sequence, the counter increments by one; otherwise, the counter remains unchanged.

Beyond the basic SNG and SNC units, the logical implementation of numerical operations is also of paramount importance in the context of SC. Given that SC involves performing computations through single-bit streams, numerous intricate operations traditionally executed in binary computing can be effectively implemented using logic gate circuits within the SC framework. As an illustration, a multiplier can be dramatically simplified to an AND gate.

**Figure 4:**
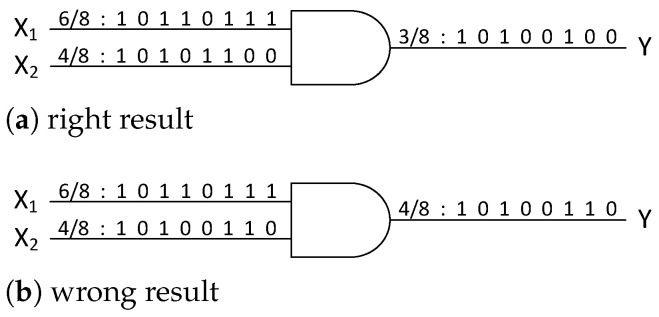
Stochastic multiplier based on AND gate.

Figure 4 shows the stochastic multiplier based on the AND gate. Consider two random bit streams, $X_{1}$ and $X_{2}$, with probability values of $\frac{6}{8}$ and $\frac{4}{8}$, respectively. When these two bit streams pass through an AND gate circuit, the output is the logical 1 only when both $X_{1}$ and $X_{2}$ are the logical 1 at the same position. Consequently, the AND gate circuit can perform stochastic multiplication. Compared to binary computing, the complexity is substantially lower in the SC multiplier. As a result, the SC offers a significant reduction in hardware resource overhead.

Figure 4 also illustrates a limitation of stochastic multipliers. The SC can only provide an approximate result, which is a random variable centered around the precise result as its expectation. As the length of the sequences increases and the correlation between the two sequences decreases, the variance in calculation will gradually diminish. Therefore, it is necessary to strike a balance between computational efficiency and precision, which is a pivotal consideration in SC design.

## 3. Hybrid Computing Architecture of MSD Algorithm

The MSD algorithm is divided into two integral components: correlation calculation and symbol decision. The correlation calculation primarily comprises two fundamental computational units: complex multiplication and complex addition. Meanwhile, symbol decision involves three essential computational units: computation of the modulus, determination of the maximum value, and the subtraction of maximum values.

For the correlation calculation, this study employs SC to reduce the complexity of implementation. Stochastic multipliers and adders exhibit lower resource consumption than binary multipliers and adders; however, compared to stochastic adders, stochastic multipliers are susceptible to the correlation of sequences. Therefore, when applying stochastic multipliers, it is necessary to ensure that the input sequences are independent. Given that complex multiplication constitutes the initial step of the MSD algorithm, the random bit sequences are directly generated by the SNGs and do not exhibit excessively high correlation. Therefore, this limitation can be ignored in the SC-based correlation calculation.

However, when utilizing SC for correlation calculation, aside from the challenge of sequence correlation, there arises a problem of data scaling. As the data range in SC is inherently limited to 0 to 1, it is imperative to maintain this range within appropriate bounds throughout the entire computation process. Stochastic multipliers, due to their inherent computational characteristics, naturally avoid this issue. Conversely, when executing stochastic addition, the use of a suitable scaling factor becomes necessary to prevent potential overflow. Both excessively large and small scaling factors can result in indistinguishable differences among various computational outcomes. Therefore, this study proposes a flexible scaling adder circuit that can select an appropriate scaling factor based on different input conditions, which can ensure that the computational results do not overflow.

When performing symbol decision in the MSD algorithm, the first step is calculating the modulus of the correlation results. If SC is adopted for this process, it requires two uncorrelated random sequences with identical probability. If a new sequence is generated based on the outcomes of the correlation calculation, it can result in higher correlation between the two sequences and larger computational errors. Therefore, binary computing is adopted to complete the symbol decision process. Therefore, in the various computational stages of the MSD algorithm, this study adopts two distinct computational paradigms—SC and binary computing—based on their respective characteristics. A hybrid computing scheme is designed to implement the hardware circuit for the entire MSD algorithm.

### 3.1. Correlation Calculation Based on SC

In the realm of SC, it is imperative to maintain probability values strictly within the range of 0 to 1 throughout the entire computation process. During multiplication operations, if the input data fall within the range of 0–1, the output will inherently lie within the same range.

For adders employed in SC, it is necessary to scale the summation result to prevent overflow. Excessively small scaling factors may lead to the summation results exceeding the permissible range, while excessively large scaling factors result in small outcomes, thereby reducing the discriminability between different summation results. Therefore, it is necessary to design the adder with an appropriate scaling factor. Additionally, considering that the input number of the adder will vary with the observation window length in the MSD algorithm, the adder must also accommodate different numbers of input ports. Thus, high requirements are imposed on the scalability and flexibility of the SC-based adders.

In summary, the entire correlation calculation based on SC requires three fundamental computational units: an inverter, a multiplier, and an adder with flexible scaling factors. The circuit structures of each basic module based on SC will be introduced individually in the following.

#### 3.1.1. Inverter

The input data are typically represented as signed numbers in the MSD algorithm. For the sake of simplifying the discussion, the input range can be limited between −1 and 1. When these input data are converted into a random bit stream by the SNG circuit, the conversion equation can be expressed as

(21) $$P_{x}=\frac{X+1}{2}$$

where *X* denotes the input data and $P_{x}$ denotes the probability data. During the mapping process from the data domain to the probability domain, the input data are scaled and a bias is added. In this scenario, to obtain the opposite number of input data, the following equation can be used:

(22) $$\begin{array}{cc} P_{\bar{x}} & =\frac{-X+1}{2}\\ \; & =1-P_{x} \end{array}$$

where $P_{\bar{x}}$ is the probability of the opposite number of *X*—that is, when $P_{x}$ is the logical 1, the $P_{\bar{x}}$ is the logical 0, and vice versa. Therefore, the computational circuit for the inverter in the SC context is a NOT gate.

#### 3.1.2. Multiplier

When signals are mapped from the data domain to the probability domain, the data undergo scaling and bias operations. To ensure uniformity in calculation, the product of two input data points must also undergo the same mapping rule. Assuming the two input data points are $X_{1}$ and $X_{2}$, the mapped result of their product can be expressed as

(23) $$P_{y}=\frac{X_{1}X_{2}+1}{2}$$

where *y* is the product of $X_{1}$ and $X_{2}$. Considering that the mapping results of input data in the probabilistic domain can be represented as

(24) $$P_{x_{1}}=\frac{X_{1}+1}{2}$$

and

(25) $$P_{x_{2}}=\frac{X_{2}+1}{2}$$

the product can be calculated as follows:

(26) $$\begin{array}{cc} P_{y} & =\frac{X_{1}X_{2}+1}{2}\\ \; & =\frac{X_{1}+1}{2}\frac{X_{2}+1}{2}+\frac{1-X_{1}}{2}\frac{1-X_{2}}{2}\\ \; & =P_{x_{1}}P_{x_{2}}+\left(1-P_{x_{1}}\right)\left(1-P_{x_{2}}\right) \end{array}$$

In probability calculations, the computation circuit structure for the multiplier is an XNOR gate.

Similarly, the complex multiplier can be expressed as

(27) $$\begin{array}{cc} P_{y} & =\frac{\frac{R\left(X_{1}\right)\times R\left(X_{2}\right)-I\left(X_{1}\right)\times I\left(X_{2}\right)}{2}+1}{2}+j\frac{\frac{R\left(X_{1}\right)\times I\left(X_{2}\right)+I\left(X_{1}\right)\times R\left(X_{2}\right)}{2}+1}{2}\\ \; & =\frac{1}{2}\left\{P_{R(x_{1})}P_{R(x_{2})}+\left(1-P_{R(x_{1})}\right)\left(1-P_{R(x_{2})}\right)\right\}-\frac{1}{2}\left\{P_{I(x_{1})}P_{I(x_{2})}+\left(1-P_{I(x_{1})}\right)\left(1-P_{I(x_{2})}\right)\right\}\\ \; & +j\frac{1}{2}\left\{P_{R(x_{1})}P_{I(x_{2})}+\left(1-P_{R(x_{1})}\right)\left(1-P_{I(x_{2})}\right)\right\}+\frac{1}{2}\left\{P_{I(x_{1})}P_{R(x_{2})}+\left(1-P_{I(x_{1})}\right)\left(1-P_{R(x_{2})}\right)\right\} \end{array}$$

where R(·) denotes the real part of a complex number and I(·) denotes the imaginary part of a complex number.

**Figure 5:**
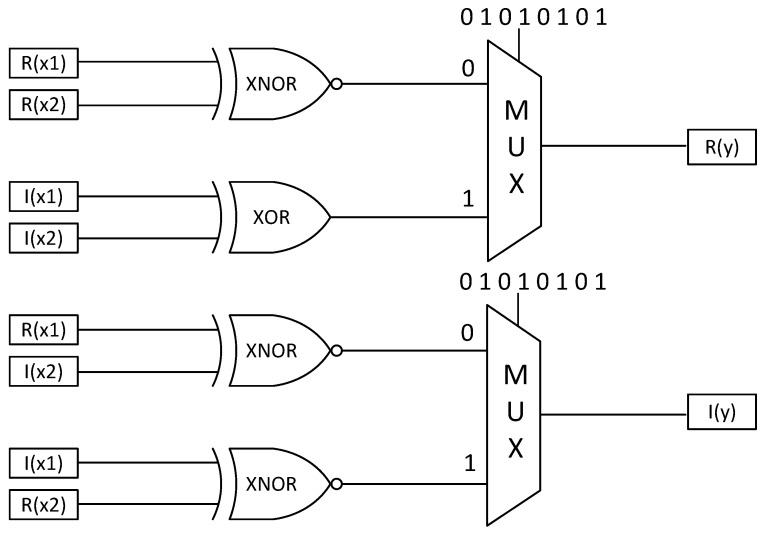
The circuit of the stochastic complex multiplier.

In the complex multiplication computation, four multipliers and two adders are required. When the input data are random, adders with a scaling factor of two should be employed to prevent result overflow. The computation circuit of $\frac{x_{1}+x_{2}}{2}$ is a two-input multiplexer in the SC. The whole circuit of the complex multiplier is shown in Figure 5.

#### 3.1.3. Adder with Flexible Scaling Factor

Assuming that the adder has N inputs and a scaling factor of k, and taking into account the scaling and bias in the domain conversion, the calculation equation for the adder can be expressed as

(28) $$\begin{array}{cc} P_{y} & =\frac{\frac{X_{1}+X_{2}+\cdots +X_{N}}{k}+1}{2}\\ \; & =\frac{1}{k}\left(\frac{X_{1}+X_{2}+\cdots +X_{N}+k}{2}\right)\\ \; & =\frac{1}{k}\left(\frac{X_{1}+1}{2}+\frac{X_{2}+1}{2}+\cdots +\frac{X_{N}+1}{2}-\frac{N-k}{2}\right)\\ \; & =\frac{1}{k}P_{x_{1}}+\frac{1}{k}P_{x_{2}}+\cdots +\frac{1}{k}P_{x_{N}}-\frac{N-k}{2k}\\ \; & =\frac{1}{k}\left(P_{x_{1}}+P_{x_{2}}+\cdots +P_{x_{k}}\right)+\frac{1}{k}\left(P_{x_{k+1}}+P_{x_{k+2}}+\cdots +P_{x_{2k}}\right)+\cdots \\ \; & +\frac{1}{k}\left(P_{x_{Mk+1}}+P_{x_{Mk+2}}+\cdots +P_{x_{N}}\right)-\frac{N-k}{2k}\\ \; & =P_{\hat{x_{0}}}+P_{\hat{x_{1}}}+\cdots +P_{\hat{x_{M}}}-\frac{N-k}{2k} \end{array}$$

where $P_{\hat{x_{i}}}=\frac{1}{k}\left(P_{x_{ik+1}}+\frac{1}{k}P_{x_{ik+2}}+\cdots +\frac{1}{k}P_{x_{ik+k}}\right)$, $M=\lfloor \frac{N}{k}\rfloor$, and ⌊·⌋ denotes the floor operation.

In the stochastic adders, when the scaling factor k is excessively large, the output becomes too small, necessitating longer random sequences to ensure the computational accuracy. For adders with N inputs, a scaling factor of up to N can guarantee that the result will not overflow. Therefore, it suffices that the scaling factor k in the adder is less than N.

As observed in Equation (28), during the stochastic addition computations, the probability value of the input data is first subjected to a scaling operation. Subsequently, as the scaled sequence is summed, a correction factor—which is dependent on both the scaling factor and the number of inputs—is subtracted from the cumulative sum.

The truth table for the adder is shown in Table 2. To simplify the discussion, the scaling process has been omitted from the table. Within the table, $P_{\frac{N-k}{2k}}$ is the random sequence with a probability value of $\frac{N-k}{2k}$, $state_{n}$ is the current state, $P_{y}$ is the output of the adder, and $state_{n+1}$ is the updated state after the output. The state register and $P_{\frac{N-k}{2k}}$ are utilized to perform the required subtraction operation.

**Figure 6:**
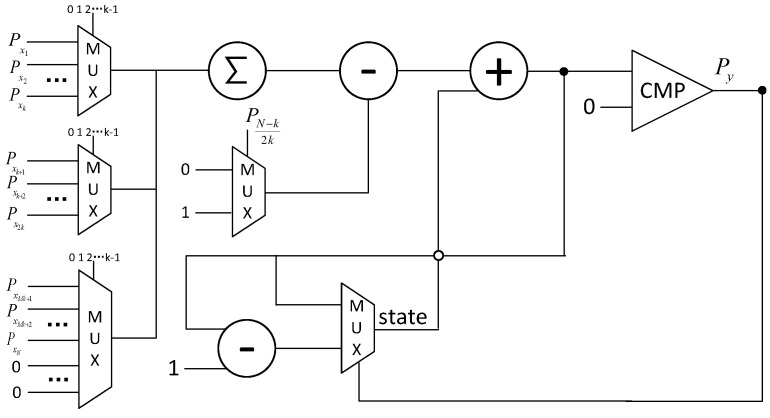
The circuit of the stochastic adder.

Figure 6 illustrates the hardware architecture of the proposed adder. In the comprehensive adder circuit, the N input signals are initially partitioned into groups of k, and a k-input multiplexer is employed to scale these signals. In the case where the last group of inputs comprises fewer than k elements, zeros are appended to ensure the completeness of the set. After scaling the input data, a subtraction unit is used to subtract the correction factor $P_{\frac{N-k}{2k}}$. Subsequently, the output of the adder is determined based on the subtraction result and the current state value. Finally, the state value is updated in accordance with the adder’s output.

When k=N, the entire adder circuit simplifies to a multiplexer. When k=1, the adder circuit constitutes a full adder. If N−k>2k, the correction factor is greater than 1. In such a case, the probabilistic value of the random sequence $P_{\frac{N-k}{2k}}$ within the adder is modified from $\frac{N-k}{2k}$ to $\frac{N-3k}{2k}$, while an extra constant decrement of 1 is applied during the subtraction computation process. As the scaling factor is generally not too small during the summation of multiple data, this particular circuit adjustment is not graphically depicted in Figure 6.

#### 3.1.4. The Correlation Circuit Architecture Based on SC

Building upon the inverters, multipliers, and adders discussed earlier, along with the integration of SNGs and SNCs, this study presents the architecture of the signal correlation circuit based on SC. Considering that the fundamental computational units within SC exhibit low resource consumption but require a long computing time to achieve acceptable computational precision, a parallel computing framework is adopted to improve the computational efficiency.

**Figure 7:**
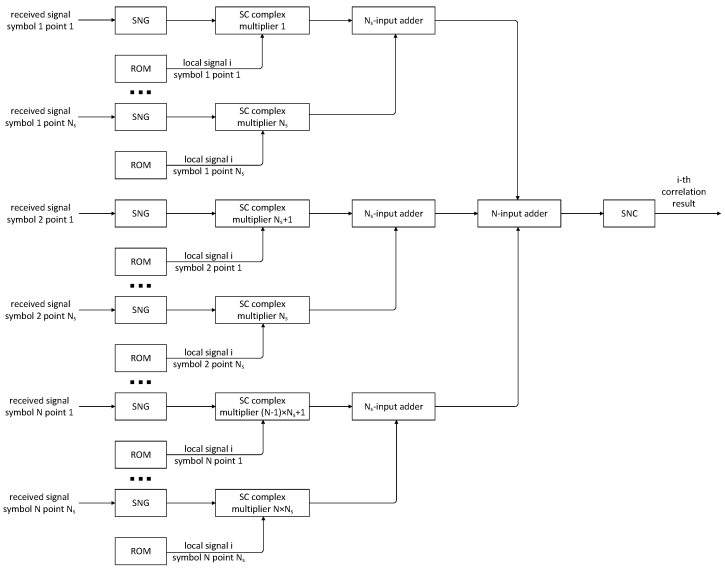
Correlation circuit based on SC.

Figure 7 illustrates the circuit diagram for the correlation calculation between the received signal and a local signal, where N is the number of symbols utilized in the MSD algorithm and $N_{s}$ is the number of sampling points per symbol. After executing complex multiplication between the received signal and local signal, a total of $N\times N_{s}$ results are obtained. This study presents a two-stage adder for this summation. The first stage sums the correlation results for each symbol, while the second stage sums the results of the N symbols again. Upon acquiring the outcomes through SC, the results should be converted into binary data using SNCs. Given that the conversion from binary data to a random sequence involves scaling and biasing operations, it is necessary to apply inverse operations on the SC results during the SNC process.

Furthermore, the local signal serves as prior information in the MSD algorithm and, thus, is directly stored as random bit sequences in the correlation circuit based on SC. On one hand, the SNGs will consume a certain amount of computational resources. On the other hand, a certain inaccuracy arises during the data domain conversion of SNGs. The pre-storage of a random sequence of the local signal serves to efficiently mitigate the computational resources while simultaneously safeguarding the accuracy of the computed results. Nevertheless, in comparison to storing the binary data of the local signal, this strategy entails a higher consumption of storage resources.

### 3.2. The Symbol Decision Pipeline Design

For the process of symbol decision, this study adopts a binary computing scheme. In the correlation calculation stage, the SC is utilized to reduce the resource consumption of hardware circuits, primarily due to the consideration that binary computing requires extensive hardware resources for complex multiplication. Nonetheless, a notable concern regarding SC is that the results of SC exhibit certain statistical errors. If the entire MSD algorithm relies solely on SC, the calculation errors will accumulate at each stage of computational unit, thereby necessitating a longer computation time to ensure the convergence of results.

Furthermore, a significant reason for employing binary computing in the symbol decision process lies in the fact that the first step involves computing the modulus of a complex signal. In binary computing, this solely necessitates the complex number itself. Conversely, the multiplier in the SC demands two uncorrelated random sequences. In the correlation calculation based on SC in the previous stage, only one random sequence can be generated. To sustain the use of SC for computing the modulus, an additional random sequence with the same probability value is necessary. When regenerating a new sequence based on an existing sequence, this may result in a certain correlation between the two sequences, thereby diminishing the computational accuracy.

Considering the challenges associated with symbol decision based on SC, reconsidering the adoption of binary computing is advisable. In comparison to SC, binary computing exhibits distinctive advantages. The errors in binary computing mainly arise from data truncation during bit quantization, and its computational accuracy is significantly higher than that of SC. Moreover, when calculating the magnitude of a signal, binary data only require self-multiplication, thereby avoiding the sequence regeneration issues encountered in SC.

Although the resource overhead of binary computing is higher than that of SC, some strategies can be employed to reduce its hardware resource utilization. It should be noted that the hardware resources in binary computing are intrinsically related to the real-time requirements of data processing. When the demand for the data processing rate is relatively low, binary computing approaches can be designed using a serial pipeline architecture, which does not incur high resource consumption. Recalling the characteristics of SC, it exhibits low hardware overhead but requires a long computation time, resulting in a relatively low update rate for correlation results. When binary computing is employed for symbol decision, its hardware resource consumption can be significantly reduced through reasonable pipeline design.

**Figure 8:**
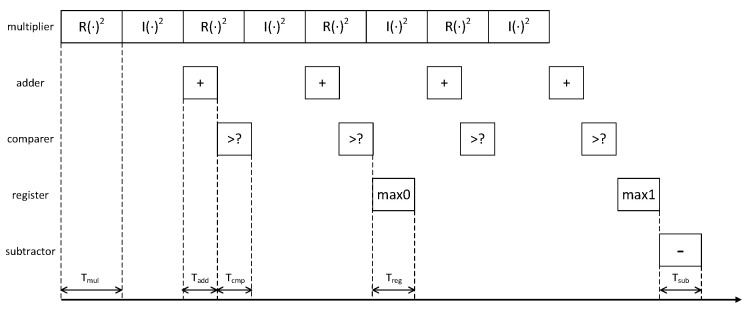
The pipeline for symbol decision.

Figure 8 illustrates the pipeline architecture for symbol decision based on binary computing. The entire pipeline comprises a multiplier, an adder, a comparator, a register, and a subtractor. The multiplier is utilized to compute the moduli of complex numbers. Given the relatively low update rate of the correlation results, this study employs a single multiplier to sequentially calculate the squares of the real and imaginary parts of the complex numbers. These values are then summed using an adder to yield the squared modulus of the complex number. Subsequently, a comparator is used to serially compare each output of the adder. Upon identifying a larger result, it is stored in the register. Once the maximum value among all correlation results corresponding to symbol 0 has been recorded, the register is reset and reused to record the maximum value among all correlation results corresponding to symbol 1. Finally, a subtractor is employed to compute the difference between these two maximum values, thereby producing the definitive symbol decision outcome. Due to the fact that the computation time $T_{mul}$ of the multiplier is greater than the computation times $T_{add}$, $T_{cmp}$, $T_{reg}$, and $T_{sub}$ of the other units, the entire pipeline scheme can be successfully implemented.

### 3.3. Multi-Stream Design for MSD Algorithm

The circuits for correlation calculation based on SC require substantial computation time to ensure the accuracy of the results, which consequently leads to a notably slower update rate for these outcomes. Meanwhile, the symbol decision based on binary computing incorporates a pipeline design to serially search for the maximum complex modulus among all correlation results, yielding a similarly low update rate for its outcomes. While the computation circuit for the entire MSD algorithm can significantly reduce hardware resource consumption, it suffers from severe limitations in terms of its real-time computation performance. Therefore, in the design of the overall MSD algorithm, a balanced consideration must be given to the trade-off between computation time and hardware resource overhead.

**Figure 9:**
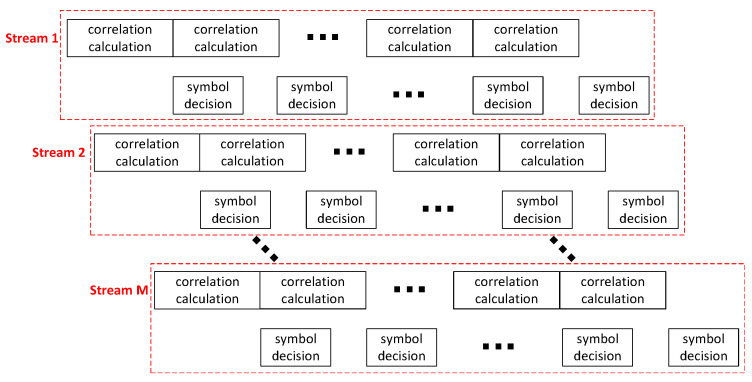
Multi-stream design for the MSD algorithm.

Figure 9 illustrates the multi-stream processing framework of the MSD algorithm. SC-based correlation calculations require hundreds of random bits to maintain acceptable error margins. Even in the 7-symbol MSD algorithm, its symbol decision only involves 128 complex numbers. The required computational time is fully accommodated within the processing time of the SC-based correlation calculation. Therefore, as shown in Figure 9, the symbol decision can be seamlessly embedded within the SC timeline without incurring extra time overhead. The inherently low data update rate of SC enables binary-based symbol decision to be implemented using pipeline processing architectures.

As the symbol decision computation time can be overlapped with the correlation computation period, the MSD algorithm enables parallel processing of these two operations. Specifically, as the correlation computation for one received signal block concludes, processing of the subsequent block immediately initiates while the symbol decision module generates output data based on the previous correlation results. However, the inherent computational latency of SC imposes limitations—even with seamless pipelining of consecutive correlation operations, the system may struggle to meet the requirements of real-time processing.

Given the low resource overhead of SC-based correlation calculations and serialized symbol decision, multi-stream parallel data processing becomes feasible. By instantiating multiple data streams, the hybrid SC-based architecture can achieve high throughput, where concurrent processing of independent data streams ensures compliance with real-time requirements.

## 4. Performance Analysis of Basic SC Unit

### 4.1. Computational Accuracy of Basic SC Unit

To ascertain the computational performance of the algorithm proposed in this paper, an initial analysis was conducted regarding the computational accuracy of the basic SC units. A total of 10,000 sets of random sequences, uniformly distributed within the range of −1 to 1, were employed as input data in order to minimize the statistical errors. The outcomes derived from floating-point computations served as the benchmark values, and the Root Mean Square Error (RMSE) was utilized to quantify and evaluate the computational performance of the various computing units.

**Figure 10:**
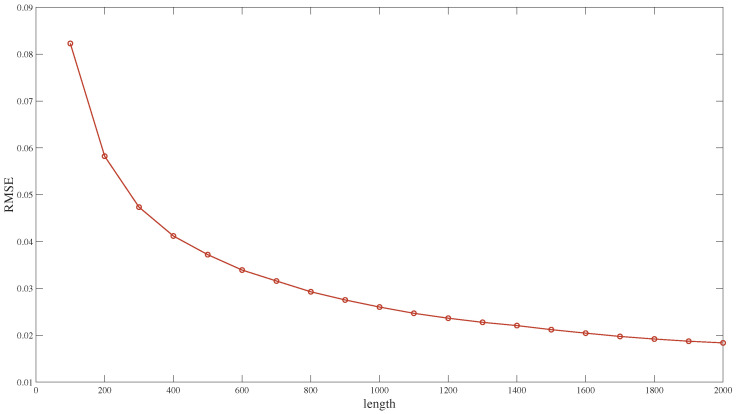
The RMSE of the inverter unit.

Figure 10 illustrates the computational accuracy of the stochastic inverter. As the inverter has only a single input and its circuit does not introduce inherent computational errors, the errors associated with the inverter stem only from the data conversion process of the SNG. As the sequence length increases, the statistical error can be reduced, leading to an enhancement in the computational accuracy of the inverter.

Distinct from the inverter, the computational results of the multiplier can be influenced by the correlation between the two input sequences. In this study, we introduced the SC correlation (SCC) methodology, as outlined in reference [30], and the corresponding calculation equation can be expressed as

(29) $$SCC\left(X,Y\right)=\left\{\begin{array}{cc} \frac{ad-bc}{n\times \min(a+b,a+c)-(a+b)(a+c)} & ifad>bc\\ \frac{ad-bc}{\left(a+b)(a+c)-n\times \max(a-d,0\right)} & otherwise \end{array}\right.$$

where *a* is the number of overlapping 1s in X and Y, *b* is the number of overlapping 1s in X and 0s in Y, *c* is the number of overlapping 0s in X and 1s in Y, and *d* is the number of overlapping 0s in X and Y. When the SCC is +1, it signifies maximum similarity between the two sequences. Conversely, when the SCC is −1, it indicates the minimum similarity (maximum difference) between the sequence and, when the SCC is 0, it implies that the two sequences are independent.

**Figure 11:**
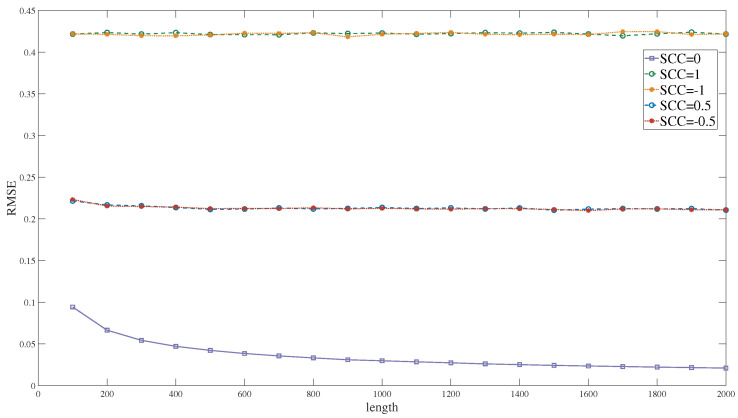
The RMSE of the multiplier unit.

Figure 11 illustrates the computational accuracy of the multiplier with input sequences exhibiting different correlation levels. When the two input sequences are independent, the computational accuracy of the multiplier progressively enhances as the sequence length increases. Under such conditions, the computational error of the multiplier primarily arises from errors during the numerical domain conversion of the two random sequences. When a notable degree of correlation is present between the two input sequences, the computational error becomes more pronounced. This error mainly stems from the deviations in the computation results due to the sequence correlation. As a result, even with an increase in the sequence length, there is generally no discernible enhancement in the computational accuracy.

**Figure 12:**
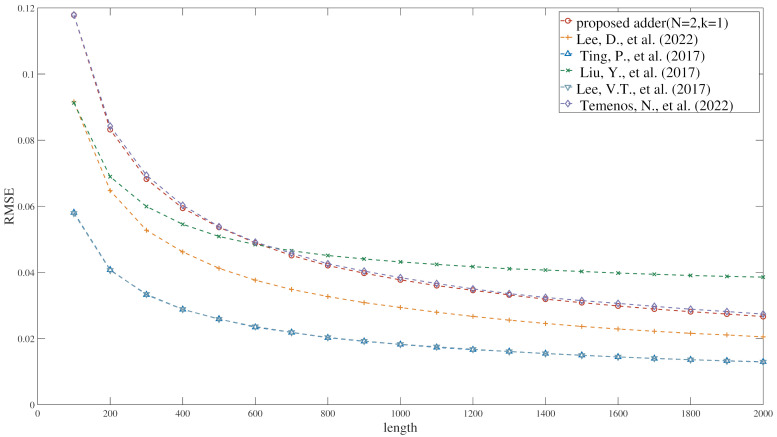
The RMSE of proposed adder and [36,37,38,39,40].

Figure 12 compares the performance of the proposed adders with those presented in references [36,37,38,39,40]. When N = 2 and k = 1, the adder proposed in this study, similar to the one presented in reference [40], is a non-scaled adder. The experimental results indicate that the computational error of the proposed adder is basically equivalent to that of reference [40]. Therefore, the proposed adder will not introduce additional computational errors. The adders described in Reference [37] and Reference [39], which also incorporate distinct methodologies to mitigate computational errors during their calculation procedures, are scaled adders with a scaling factor of one half. The computational error of the proposed adder is slightly higher than that of the adders in Reference [37] and Reference [39].

**Figure 13:**
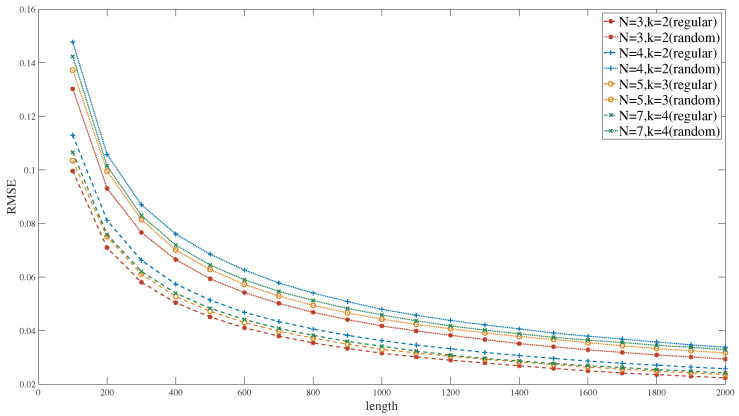
The RMSE of the proposed adder under different configurations.

Figure 13 illustrates the computational accuracy under diverse configurations of the proposed adder with different reference sequences. The results indicate that, regardless of variations in the adder’s input count N and scaling factor k, the computational errors remain essentially the same, with no significant distinctions. Consequently, the adder proposed in this study demonstrates remarkable flexibility without any compromise in computational accuracy.

Additionally, during the computation process, the proposed adder requires the subtraction of a random sequence with a constant probability value. Experimental evaluations were conducted to assess the computational accuracy when integrated with two distinct types of reference sequences: regular sequences (e.g., 0101 or 001001) and random sequences generated by the SNGs. The figure shows that the adder based on regular sequences demonstrated higher computational accuracy, which can primarily be attributed to the inherent errors within random sequences generated by the SNGs.

Therefore, the adder architecture adopted in this study eliminates the need for supplementary SNGs, instead leveraging direct and regular reference sequences to enhance its performance. This approach not only simplifies the system design but also significantly reduces the overall resource consumption, thereby obtaining a substantial improvement in computational efficiency.

### 4.2. Hardware Resources of Basic SC Unit

The significant advantage of SC is its relatively low consumption of hardware resources. This study conducted experiments to assess the hardware resource overhead of different SC units based on a Field-Programmable Gate Array (FPGA) platform.

Table 3 illustrates the resource overhead of the inverters and multipliers based on SC and binary computing. The hardware resource of stochastic inverters is approximately one-tenth that of binary inverters, and the stochastic multipliers consume approximately 1% compared to their binary implementations. Furthermore, the resource consumption of stochastic complex multipliers is significantly less than that under binary computing. Consequently, this study adopted SC for the correlation calculation in the MSD algorithm.

Table 4 illustrates the hardware resource overhead of the proposed adder, in comparison with the stochastic adders in References [36,37,38,39,40] and binary adders. In comparison to both the stochastic non-scaled adder [38,40] and the binary adder, the proposed adder demonstrated a slightly lower resource overhead. However, when compared to the scaled adder presented in the literature [36,37,39], the proposed adder achieved non-scaled operations at the cost of a marginally increased resource overhead.

Table 5 illustrates the hardware resource overhead of both stochastic adders and binary adders under different input numbers. The table reveals that the resource consumption of the stochastic adders does not significantly increase with the number of input ports. Additionally, stochastic adders demonstrate a lower resource consumption compared to binary adders, indicating their advantages for hardware implementation.

## 5. Performance Analysis of MSD Algorithm Based on Hybrid SC

### 5.1. Computational Accuracy of MSD Algorithm

In order to assess the performance of the MSD circuit constructed using the hybrid SC approach, this study initially carried out an experiment to evaluate the overall computational accuracy of the MSD algorithm. The sampling factor of the receiver was set to four. Leveraging the previously designed fundamental SC units, the computational accuracy was verified for the three-, five-, and seven-symbol variants of the MSD algorithm.

Additionally, the experiments also involved comparing the computational accuracy of the hybrid SC approach proposed in this study with that of a pure SC approach; specifically, the pure SC method employed a re-randomization technique in the symbol decision stage, as outlined in [41].

**Figure 14:**
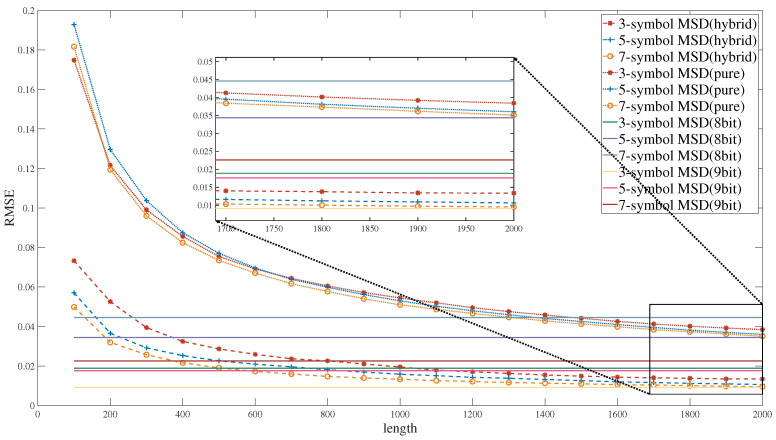
The RMSE of MSD algorithm variants.

Figure 14 illustrates the computational accuracy across diverse computation schemes and different symbol lengths. The results show that the hybrid SC achieved higher computational accuracy than pure SC. This can be attributed to two primary reasons: First, although re-randomization allows a new random sequence to be regenerated based on an existing random sequence, it does not fully eliminate the correlation between the two sequences. Second, re-randomization largely maintains the probability values of the sequences. When computational errors exist in the sequences undergoing re-randomization, these errors are propagated to the newly generated sequences, resulting in increased computational errors. In light of these observations, this study adopts a hybrid SC method to develop the MSD algorithm, aiming to enhance computational accuracy while ensuring computational efficiency.

Figure 14 also illustrates the computational accuracy under different bit quantization schemes. The error in binary computation arises from two primary sources: (1) quantization loss determined by the bit width; (2) the scaling operations that are necessary to prevent data overflow during summation processes. As the observation window of the MSD algorithm expands from three to seven symbols, the summation of input data increases proportionally. This necessitates a larger scaling factor to prevent overflow in adder operations, which introduces greater computational errors. Consequently, the seven-symbol MSD exhibited a greater computational error than the three-symbol MSD.

Figure 14 provides a comparative analysis of computational accuracy between hybrid SC and binary computing paradigms. However, it is critical to recognize that error manifestations differ fundamentally between these architectures: Binary computation introduces errors through floor operations, while the errors from hybrid SC derive from inherent variance in probabilistic bit-stream representations. Consequently, direct numerical comparison of error metrics does not provide an equitable reflection of algorithmic performance across different computational frameworks.

### 5.2. BER Performance of MSD Algorithm

In the MSD algorithm, the output result is determined based on the maximum correlation value derived from comparisons between the received signal and a set of local signals, distinguished by their symbols being either zero or one. The performance of the MSD cannot be adequately represented solely according to the computational error. Therefore, this study evaluates the BER performance under three-symbol, five-symbol, and seven-symbol MSD algorithms based on hybrid SC.

**Figure 15:**
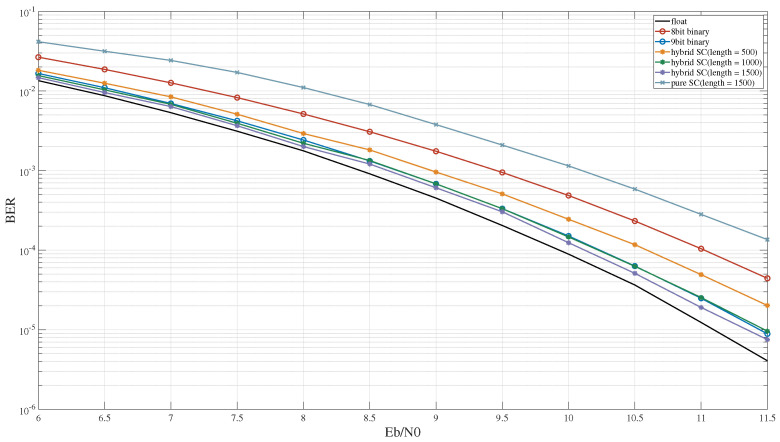
The BER performance of the 3-symbol MSD algorithm.

Figure 15 illustrates the BER performance of the three-symbol MSD algorithm under different computational schemes and sequence lengths. For a sequence length of 500 in hybrid SC, the BER performance slightly surpassed that with 8-bit binary computing. As the sequence length increased to 1000, the BER performance of hybrid SC was comparable to that of 9-bit binary computing. Additionally, when compared to hybrid SC, the MSD algorithm experienced a more pronounced performance decrease under pure SC.

**Figure 16:**
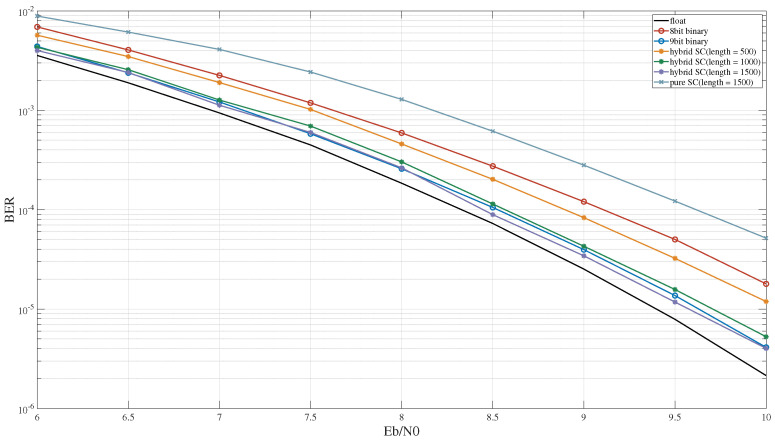
The BER performance of the 5-symbol MSD algorithm.

Figure 16 illustrates the BER performance of the five-symbol MSD algorithm under different conditions. Similar to the three-symbol MSD, when the sequence length was set to 500, hybrid SC demonstrated a marginally superior BER performance compared to 8-bit binary computing. However, to attain BER performance equivalent to 9-bit binary computing, the sequence length for hybrid SC must be extended to 1500. This is primarily due to the fact that, as the number of symbols in the MSD algorithm increases, the performance loss of binary computing under the same bit quantization becomes smaller. The loss in binary computing mainly stems from data truncation. As the number of symbols in the MSD algorithm increases, the number of potential outcomes of the symbol decision process expands, thereby mitigating the adverse effects of quantization loss. Conversely, the primary error source in hybrid SC arises from the fluctuation of its computation results, rendering it relatively insensitive to the number of symbols in the MSD algorithm.

**Figure 17:**
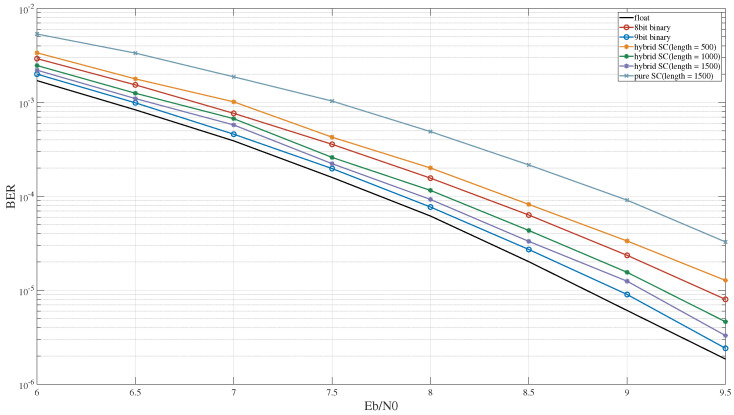
The BER performance of the 7-symbol MSD algorithm.

Figure 17 illustrates the BER performance of the seven-symbol MSD algorithm under different conditions. Compared to those of the three- or five-symbol MSD algorithms, the hybrid SC in the seven-symbol MSD necessitated a longer sequence length to sustain its BER performance at a comparable level to binary computing. When the sequence length reached 1000, the BER performance of hybrid SC marginally exceeded that of 8-bit binary computing. However, even when the sequence length extended to 1500, the performance of hybrid SC remained slightly inferior to that of 9-bit binary computing.

### 5.3. Hardware Resource Overhead of MSD Algorithm

To assess the hardware resource overhead of the hybrid SC approach designed in this study, the entire hardware circuit was meticulously implemented on the FPGA platform. Specifically, the Xilinx Virtex UltraScale xcvu190 platform was utilized, and the synthesis and implementation tools were Vivado Synthesis 2018 and Vivado Implementation 2018, respectively. As previously mentioned, a multi-stream approach was adopted to balance hardware resource consumption and computing speed. Due to the variation in BER performance under different sequence lengths in hybrid SC, this study conducted a precise evaluation of hardware resource consumption for various sequence lengths through controlling the number of parallel-processed streams. Considering actual data processing requirements, the symbol rate of the transmitted data was set to 10 Msps, and the up-sampling factor of the receiver was set to four. For comparison, resource requirements for binary computing under 8- and 9-bit quantization were also established.

Due to the up-sampling factor, a four-input adder is required to accumulate the multiplication results within each symbol. For the three-, five-, and seven-symbol MSD algorithms, this architecture necessitates three-, five-, and seven-input adders, respectively, to perform the summation of accumulated results across multiple symbols.

To determine the optimal scaling factors for each adder, we conducted numerical simulations of the MSD algorithm under the constraint that the summation results must not exceed the limiting range while minimizing the scaling factor. Through numerical analysis of the MSD computational process, we established the following scaling factor assignments: two for three-input adders, two for four-input adders, three for five-input adders, and four for seven-input adders. The selection criteria aimed to balance between preventing overflow in addition operations while preserving maximum signal resolution through minimal scaling factors.

Table 6 illustrates the hardware resource overhead of the three-symbol MSD algorithm under different computing paradigms. When the sequence length was 500, the hybrid SC proposed in this study exhibited a lower hardware resource consumption than that demanded by 8-bit binary computing. When the sequence length reached 1000, the hardware resource consumption of hybrid SC surpassed that of 8-bit binary computing, yet remained inferior to that of 9-bit binary computing. As the sequence length exceeded 1500, the hardware resource consumption of hybrid SC surpassed the threshold for 9-bit binary computing.

Table 7 illustrates the hardware resource overhead of the five-symbol MSD algorithm. In comparison to the three-symbol MSD, the hybrid SC demonstrated a hardware resource consumption that remained slightly lower than that of 8-bit binary computing, even when the sequence length reached 1000. When the sequence length increased to 1500, the hardware resource consumption of hybrid SC exceeded that of 8-bit binary computing but remained less than that of 9-bit binary computing. This is primarily due to the non-linear relationship between hardware resource consumption and computational load in SC. Even though an increase in the number of MSD symbols leads to a linear increase in computational load, the resource consumption does not increase significantly.

Table 8 illustrates the hardware resource overhead of the seven-symbol MSD algorithm. When the sequence length reached 1500, the hardware resource of hybrid SC slightly exceeded that of 8-bit binary computing. Conversely, when the sequence length was reduced to 500, the hardware resource of hybrid SC was merely one-third that with 8-bit binary computing. Nonetheless, the utilization of storage resources in hybrid SC exhibited a notably higher consumption compared to binary computing. This is primarily due to the fact that hybrid SC stores random bit sequences of local signals, which mitigates the computing resource overhead for numeric domain conversion at the cost of additional storage resources. Considering the ample storage capacity in modern hardware systems, this increased storage requirement can be considered an acceptable compromise.

### 5.4. Power Consumption and Computational Latency Analysis

In addition to comparing the hardware resource overheads, this paper also compares the power consumption and computational latency under the hybrid SC and the binary computation scheme.

Table 9 illustrates the power consumption of different computing paradigm. When the sequence length is 500, energy consumption of hybrid SC is lower than that of binary computing. However, when the sequence length exceeds 1000, energy consumption of hybrid SC is greater. Compared with its advantages in resource overhead, the energy efficiency of hybrid SC is slightly lower. Nevertheless, when the sequence length is short, it can still reduce energy consumption while reducing resource overhead.

Table 9 also illustrates the computational latency of different computing paradigm. Compared with binary computation, the computational latency of hybrid SC is significantly increased. As the sequence length increases, the computational latency also increases linearly. But even when the sequence length is 1500, its computational latency is less than 10 microseconds. This latency is of negligible significance in the communication systems.

## 6. Conclusions

This study proposed a novel hardware framework for the MSD algorithm based on a hybrid SC approach. For the correlation calculation in the MSD algorithm, the SC scheme is employed to reduce the hardware resource overhead. In the SC-based correlation calculation, a flexible and scalable stochastic adder was developed and integrated to achieve summation operations using different scaling factors. For the binary computing-based symbol decision, a pipeline structure was proposed to execute the entire process serially, thus reducing the resource overhead by leveraging the low update rate of the SC-based correlation results. The hardware architecture based on hybrid SC for three-, five-, and seven-symbol MSD algorithm variants was successfully constructed on an FPGA platform. Experimental evaluations revealed that the BER performance of the hybrid SC is comparable to that when using traditional binary computing methods, while simultaneously achieving a substantial reduction in hardware resource utilization.

However, the hardware architecture based on hybrid SC also has some drawbacks. Its energy cost increases slightly, and computational latency is relatively high. Despite the drawbacks, the introduction of SC still has significant advantages in reducing resource overhead. In future work, it is advisable to consider applying it to other algorithms with high computational complexity.

## Figures and Tables

**Table 1:** The computational complexity of the MSD algorithm.

Observation Window of MSD Algorithm	3-Symbol	5-Symbol	7-Symbol
Number of real multiplications	400 N	2624 N	14,592 N
Number of real additions	376 N	2528 N	14,208 N

**Table 2:** Truth table of adder.

$P_{\frac{N-k}{2k}}$	$P_{\hat{x_{0}}}P_{\hat{x_{1}}}\cdots P_{\hat{x_{M}}}$	${\mathrm{state}}_{n}$	$P_{y}$	${\mathrm{state}}_{n+1}$
0	0 logical `1’s	n	n>0?1:0	n>0?n−1:n
1	0 logical `1’s	n	n>1?1:0	n>1?n−2:n−1
0	1 logical `1’	n	n>−1?1:0	n>−1?n:n+1
1	1 logical `1’	n	n>0?1:0	n>0?n−1:n
0	2 logical `1’s	n	n>−2?1:0	n>−2?n+1:n+2
1	2 logical `1’s	n	n>−1?1:0	n>−1?n:n+1
…	…	…	…	…
0	M logical `1’s	n	n>−M?1:0	n>−M?n+M−1:n+M
1	M logical `1’s	n	n>−M+1?1:0	n>−M+1?n+M−2:n+M−1
0	M + 1 logical `1’s	n	n>−M−1?1:0	n>−M−1?n+M:n+M+1
1	M + 1 logical `1’s	n	n>−M?1:0	n>−M?n+M−1:n+M

**Table 3:** Hardware resource overhead of inverters and multipliers.

Basic Unit	Inverter			Real Multiplier			Complex Multiplier		
	SC	8-Bit	9-Bit	SC	8-Bit	9-Bit	SC	8-Bit	9-Bit
LUT	1	6	7	1	72	90	2	297	366
FF	1	8	9	1	66	117	2	252	431

**Table 4:** Hardware resource overhead of 2-input adders.

2-Input Adder	Proposed	[36]	[37]	[38]	[39]	[40]	8-Bit	9-Bit
LUT	4	2	3	5	3	5	5	5
FF	4	1	3	7	3	5	8	9

**Table 5:** Hardware resource overhead of different adders.

Adder	3-Input Adder			4-Input Adder			5-Input Adder			7-Input Adder		
	k = 2	8-Bit	9-Bit	k = 2	8-Bit	9-Bit	k = 3	8-Bit	9-Bit	k = 4	8-Bit	9-Bit
LUT	6	23	26	6	29	32	7	45	51	7	59	66
FF	6	27	30	6	28	31	6	54	60	6	64	71

**Table 6:** Hardware resource overhead of 3-symbol MSD.

3-Symbol MSD	Hybrid SC (Length = 500)	Hybrid SC (Length = 1000)	Hybrid SC (Length = 1500)	8-Bit Binary	9-Bit Binary
LUT	10,631 (54%)	20,614 (105%)	30,441 (155%)	19,585 (100%)	23,582 (120%)
FF	13,846 (69%)	26,860 (134%)	40,344 (201%)	20,050 (100%)	30,315 (151%)
BRAM	2 (200%)	2 (200%)	2 (200%)	1 (100%)	1 (100%)

**Table 7:** Hardware resource overhead of 5-symbol MSD.

5-Symbol MSD	Hybrid SC (Length = 500)	Hybrid SC (Length = 1000)	Hybrid SC (Length = 1500)	8-Bit Binary	9-Bit Binary
LUT	50,577 (40%)	100,336 (79%)	144,929 (114%)	127,325 (100%)	153,194 (120%)
FF	58,604 (45%)	117,173 (90%)	175,140 (135%)	129,732 (100%)	195,827 (151%)
BRAM	4 (400%)	8 (800%)	12 (1200%)	1 (100%)	1 (100%)

**Table 8:** Hardware resource overhead of 7-symbol MSD.

7-Symbol MSD	Hybrid SC (Length = 500)	Hybrid SC (Length = 1000)	Hybrid SC (Length = 1500)	8-Bit Binary	9-Bit Binary
LUT	249,464 (35%)	501,631 (71%)	751,951 (107%)	703,214 (100%)	847,415 (121%)
FF	257,363 (36%)	514,703 (72%)	771,395 (108%)	714,168 (100%)	1,080,104 ( 151%)
BRAM	20 (1000%)	40 (2000%)	60 (3000%)	2 (100%)	2 (100%)

**Table 9:** Power consumption and computational latency of different computing paradigms.

MSD	Computing Scheme		Power Consumption	Computational Latency
3-symbol	hybrid SC	length = 500	0.76 W (76%)	2.08 μs (2600%)
		length = 1000	1.41 W (141%)	4.08 μs (5100%)
		length = 1500	1.99 W (199%)	6.08 μs (7600%)
	binary	8-bit	0.99 W (100%)	0.08 μs (100%)
		9-bit	1.30 W (131%)	0.08 μs (100%)
5-symbol	hybrid SC	length = 500	3.98 W (72%)	2.27 μs (2838%)
		length = 1000	7.73 W (139%)	4.27 μs (5338%)
		length = 1500	11.90 W (215%)	6.27 μs (7838%)
	binary	8-bit	5.55 W (100%)	0.08 μs (100%)
		9-bit	7.12 W (128%)	0.09 μs (113%)
7-symbol	hybrid SC	length = 500	20.85 W (69%)	3.04 μs (3378%)
		length = 1000	32.23 W (107%)	5.04 μs (5600%)
		length = 1500	51.83 W (172%)	7.04 μs (7822%)
	binary	8-bit	30.11 W (100%)	0.09 μs (100%)
		9-bit	38.61 W (128%)	0.10 μs (111%)

## Data Availability

The original contributions presented in this study are included in the article. Further inquiries can be directed to the corresponding author.
